# Prevalence of dementia diagnoses not otherwise specified in eight European countries: a cross-sectional cohort study

**DOI:** 10.1186/s12877-019-1174-3

**Published:** 2019-06-24

**Authors:** Connie Lethin, Ingalill Rahm Hallberg, Anna Renom Guiteras, Hilde Verbeek, Kai Saks, Minna Stolt, Adelaida Zabalegui, Maria Soto-Martin, Christer Nilsson

**Affiliations:** 1 Faculty of Medicine, Department of Health Sciences, Lund University, Box 157, 221 00 Lund, Sweden; 2 Clinical Memory Research Unit, Department of Clinical Sciences, Lund University, Jan Waldenströms gata 35, Malmö, Sweden; 3 University Hospital Parc de Salut Mar, Passeig Marítim 25-29, 08003 Barcelona, Spain; 4 Department of Health Services Research, CAPHRI, Faculty of Health, Medicine and Life Sciences, Maastricht University, P.O. Box 616, 6200 MD Maastricht, the Netherlands; 5 Department of Internal Medicine, University of Tartu, L. Puusepa 8, 50406 Tartu, Estonia; 6 Department of Nursing Science, University of Turku, FI-20014 Turun yliopisto, and Turku University Hospital, Kinakvarngatan 4-8, 20521 Turku, Finland; 7 Hospital Clinic of Barcelona, Villarroel, 170, 08036 Barcelona, Spain; 8 Department of Geriatric Medicine, Gerontopole, Toulouse University Hospital, INSERM 1027, Toulouse, France

**Keywords:** Dementia, Diagnosis, Geriatrics, Home care, Nursing homes, Ordinary housing, Neurocognitive disorders, Regression analysis

## Abstract

**Background:**

Dementia is a syndrome, with a wide range of symptoms. It is important to have a timely diagnosis during the disease course to reduce the risk of medication errors, enable future care planning for the patient and their relatives thereby optimizing quality of life (QoL). For this reason, it is important to avoid a diagnosis of dementia not otherwise specified (DNOS) and instead obtain a diagnosis that reflects the underlying pathology. The aim of this study was to investigate the prevalence and associated factors of DNOS in persons with dementia living at home or in a nursing home.

**Methods:**

This is a cross-sectional cohort study performed in eight European countries. Persons with dementia aged ≥65 years living at home (*n* = 1223) or in a nursing home (*n* = 790) were included. Data were collected through personal interviews with questionnaires based on standardised instruments. Specific factors investigated were sociodemographic factors, cognitive function, and mental health, physical health, QoL, resource utilization and medication. Bivariate and backward stepwise multivariate regression analyses were performed.

**Results:**

The prevalence of DNOS in the eight participating European countries was 16% (range 1–30%) in persons living at home and 21% (range 1–43%) in persons living in a nursing home. These people are more often older compared to those with a specific dementia diagnosis. In both persons living at home and persons living in a nursing home, DNOS was associated with more severe neuropsychiatric symptoms and less use of anti-dementia medication. In addition, persons with DNOS living at home had more symptoms of depression and less use of antidepressant medication.

**Conclusions:**

The prevalence of DNOS diagnosis is common and seems to vary between European countries. People with DNOS are more often older with more severe neuropsychiatric symptoms and receive fewer anti-dementia medication, anxiolytics and antidepressants. This would support the suggestion that a proper and specific diagnosis of dementia could help the management of their disease.

## Background

The code “dementia not otherwise specified (DNOS)” is used when symptoms and findings of cognitive dysfunction do not meet the criteria for a specific type of dementia. It may be used when the cause of dementia is unknown despite investigation [1] or when the interpretation of findings depends on the experience and specialization of the investigating physician. A specific dementia diagnosis should be timely and made when older people and their families are raising their concerns, rather than screening older people proactively for early signs and symptoms [2]. People with dementia caused by neurodegenerative diseases such as Alzheimer’s disease (AD) or Lewy body dementia (LBD) and a specific range of cognitive decline may benefit from treatment with acetylcholine esterase inhibitors [3] and memantine [4] if tolerated [5], while the latter has no effect and may even worsen behavioural and cognitive symptoms in frontotemporal dementia (FTD) [6]. Timely diagnosis and treatment with acetylcholine esterase inhibitors may improve memory and concentration as well as independence in activities in daily living (ADLs) [7, 8] and in turn improve quality of life (QoL) [9]. In addition, a diagnosis is an important aspect of the care pathway for people with dementia and their caregivers. When provided early in the disease process it allows for future planning [10], if later it is still useful as it enables access to services and it provides explanation regarding behaviours and illness outcomes which caregivers and people with dementia have identified as beneficial. This in turn may result in the possibility for the person to live in ordinary housing and delay nursing home admission [11].

The most common dementia diagnosis worldwide is AD (50–70%), followed by vascular dementia (VaD) (20–30%), FTD (5–10%) and LBD (5%). It is also common for AD to occur together with VaD [10, 12]. The diagnosis “DNOS” has been shown to be a common dementia diagnosis (43%) depending on geographical location [13]. The prevalence of DNOS has been found to be 19% for persons living in assisted living in the United States [14], and 27% for residents living in nursing homes in Sweden [15]. In European countries, research regarding the prevalence of DNOS, in people living at home or in a nursing home and the relation of DNOS to comorbidity, cognitive function, QoL, ADLs, and depression is to our knowledge scarce. In Europe the diagnosis of dementia can be made in outpatient care (primary care), in private clinics or in specialized memory clinics depending on the health care system and the structure of reimbursement [10]. In one meta-analysis, undiagnosed dementia was reported to be 61,7% worldwide, with a lower rate in Europe and North America (vs China and India) [16]. A previous study by Butler et al. [13] reported that persons who visited a geriatrician or neurologist were more likely to have a specific dementia diagnosis. Furthermore, the study showed that the use of the term “DNOS” was lowest in centres where the majority of physicians investigating persons with cognitive impairment were specialized in dementia disorders. Specialists performed cognitive testing in 98% of patients, compared with only 12% for general practitioners (GP) even though 42.5% met the criteria for a specific dementia diagnosis [13].

Factors contributing to diagnosis of dementia involve health care providers, and patient, caregiver and system factors [17]. Bond et al. [18] reported that GP’s do not recognize or are not aware of early symptoms in AD and therefore fail to diagnose the disease. In their study, the average delay from symptom onset was 20 months (range 10–32 months). Furthermore, the average time for carers to consult a physician from recognition of symptoms was 47 weeks due to lack of awareness, recognition of symptoms, believing it was a medical condition or denial. Governmental indifference to AD patients and caregivers was also believed to exist [18]. A proactive approach to diagnosis may improve QoL and timely diagnosis may provide time for the person with dementia and their family to prepare for future care and contribute to the process of care planning [16, 19]. This may also be significant for both the person and the formal and informal caregivers in understanding and how to handle problems like depression and behavioural and psychological symptoms of dementia (BPSD).

There is no cure to dementia diseases and pharmacological and non-pharmacological treatments are symptomatic and aim to maintain functional ability and independence of the person, as well as QoL and well-being [10, 19]. Acetylcholinesterase inhibitors are used for the treatment of AD and LBD if tolerated and in some cases supplemented with memantine. However, adverse effects such as nausea, vomiting, falls, bradycardia (donepezil) and deaths have been reported [5]. Acetylcholinesterase inhibitors are approved in most European countries but with differences regarding access to pharmacological treatment due to prescribing practices, reimbursement and health care systems. In some European countries treatment decisions have to be made in specialist centres and by specialist doctors, while others require continuous evaluation by the specialist responsible for the treatment decision [10]. Acetylcholinesterase inhibitors and memantine are believed to ameliorate BPSD but the evidence is contradictory since BPSD may vary due to the type of dementia and the stage of the dementia disease [20]. Although BPSD are often treated with antipsychotics, these should be avoided or used cautiously due to common adverse effects, especially in older people [21]. Often they are seen as potentially inappropriate medications in this population [22]. In addition, antidepressants have been shown to be modestly effective for behavioural symptoms such as agitation [23].

A timely dementia diagnosis is the key to medical treatment, care and support [10, 24] and may delay nursing home admission [11]. Doctors should be aware that DNOS or lack of diagnosis of dementia impacts accurate medical care, access to available services and QoL for both the person with dementia and their caregiver. Improving the timeliness of the diagnosis will also enable the opportunity for treating any non-dementia illness impacting cognition, assessment of suitability for any anti-dementia medication, and for involving the person with dementia in decision making regarding the future. To understand the complexity of DNOS, the aim of this study was to investigate the prevalence of DNOS in persons with dementia ≥65 years of age, living at home or living in a nursing home in eight European countries, and to investigate factors associated with DNOS regarding demographics, comorbidity, cognitive function, QoL, ADLs, depression and resource utilization in order to get a broader understanding of DNOS and the required treatment. Furthermore, we aimed to investigate factors associated with DNOS regarding behavioural symptoms and psychotropic medication use.

## Methods

### Study design, setting and participants

This study was a cross-sectional cohort study performed 2010–2013 in eight European countries within the European project RightTimePlaceCare (RTPC) (funded under the EU 7th Framework Programme for Research, contract number 24 21 53). Participating countries were Estonia, Finland, France, Germany, the Netherlands, Spain, Sweden and the United Kingdom [25]. The eight countries have different welfare systems and health care and social service system with regard to the responsibility for the family. In continental Europe, the role of the family is most important, in the Nordic countries, the state has a central role and in the Anglo-Saxon countries commercial caregivers predominate. In this study, screening for dementia (not available in Estonia) was mainly performed by GPs and in some countries also by registered nurses (Finland, Spain and the United Kingdom). Professionals in this study involved in the procedures to establish the diagnosis, pharmacological treatment for dementia and pharmacological treatment for BPSD were GPs and in some cases medical specialists in neurology, psychiatry or geriatrics [26]. The study population consisted of 2013 persons with dementia, 1223 living at home and 790 living in a nursing home in both rural and urban areas. Inclusion criteria were age ≥ 65 years, having a primary dementia diagnosis, having a Standardized Mini-Mental State Examination (SMMSE) score ≤ 24 [27], and living at home with support from formal health care and social services and at risk of moving to a nursing home within 6 months, or living in a nursing home. The diagnoses of people with dementia were established in outpatient care by GPs or specialist physicians (neurologists, geriatricians, psychiatrists) or in specialized memory clinics. Recruitment of participants was through a contact person in each country and the same procedure as described previously [25] was followed. Participants were recruited in outpatient care and nursing homes, and in inpatient care in hospitals and psychogeriatric clinics. Data were collected through personal interviews with questionnaires based on standardised instruments outlined below. Demographic data collected for this study was age and gender of the person with dementia, as well as duration of symptoms in years and dementia diagnosis. Factors related to DNOS included cognition, comorbidity, QoL, ADLs, depression, resource utilization and behavioural problems. Furthermore, to investigate pharmacological treatment, information using Anatomical Therapeutic Chemical Classification code was collected regarding the use of psychotropics and antipsychotics (ATC N05A), anxiolytic (ATC N05B), sedatives (ATC N05C), antidepressants (ATC N06A) and anti-dementia medication (ATC N06D).

### Measurements

The main variable for this study, DNOS, was obtained from the questionnaire on which the interviewer entered type of dementia as AD, AD/VaD, VaD, FTD, LBD, unknown or other diagnosis. Unknown diagnosis was considered as DNOS. Comorbidity was measured with the Charlson Comorbidity Index (CCI) [28]. To measure cognition, the SMMSE [27] was used. QoL in persons with dementia was measured using the Quality of life in Alzheimer’s disease (QoL-AD) [29], and ADLs were measured using the Katz Index of Independence in Activities of Daily Living (Katz-ADL) [30]. Depression was measured using the Cornell Scale for Depression in Dementia (CSDD) [31] and resource utilization regarding hospital admission and visiting a physician was measured using the Resource Utilization in Dementia questionnaire (RUD) [32]. Further, BPSD were measured with the Neuropsychiatric Inventory Questionnaire (NPI-Q) [33] to rate the severity of symptoms of the person with dementia. Measurements have been described in detail elsewhere [25]. An external audit of data was performed in each country to ensure the best quality of data collection (regarding data plausibility and management) and covered at least 20% of randomly selected records. The auditor also visited at least one or two participating institutional nursing care facilities and home care organizations to verify participation in the study.

### Analysis

For the association analysis in the RTPC study, inclusion of 785 participants was needed and for mean differences, 393 participants was needed to demonstrate effect size with a power of 80% (α = 0.05). In home care, drop-out rate was expected to be 15% and consequently 175 informal caregivers and persons with dementia dyads per country were planned to be included with a total of 1400 dyads. In this study, the following characteristics of participants with DNOS in eight European countries are described: demographics, comorbidity, cognitive function and QoL. Furthermore, the study includes ADLs, depression, resource utilization, BPSD and medical treatment. For missing data in both the SMMSE and the CCI, handling of missing data was not applicable, i.e. no summary score was calculated. For QoL-AD, a maximum of two missing items were replaced with the mean score. For the NPI and the Katz ADL, no missing values were imputed. For the Katz ADL, no total score was calculated if one item was missing. Mean score was imputed in the CSDD with a maximum of three missing items. No total score was calculated if more than three items were missing. The answer option “unable to evaluate” was treated as a missing value. For regression analysis, the dependent variable type of dementia was dichotomized into “DNOS” =1 and other dementia diagnosis = 0 (AD, AD/VaD, VaD, FTD, LDB and “other diagnosis”). For the independent variables (CCI, SMMSE, QoL-AD, Katz-ADL, CSDD, and NPI-Q scores, and psychotropic medication; antipsychotics; anxiolytic; sedatives; antidepressants; and anti-dementia medication including acetylcholine esterase inhibitors and memantine) bivariate regression analysis was performed for DNOS when living at home or in a nursing home. To analyse the association between DNOS and the independent variables, backward stepwise multivariate regression analyses (living at home/living in a nursing home) were performed and *p* = ≤0.05 was regarded as significant. All variables used in the bivariate regression analysis were also used for the multivariate regression analysis. For the analysis, SPSS version 25.0 of was used (IBM Corporation, Armonk, NY, USA).

## Results

### Prevalence of DNOS

The prevalence of DNOS in the eight participating European countries was 16% in persons living at home and 21% in persons living in a nursing home (Table 1). The prevalence of DNOS in those living at home was highest in the Netherlands (30%) and lowest in Finland (1%) (Table 2). Regarding those living in nursing homes, DNOS was most frequent in Germany (43%) and least frequent in the United Kingdom (7%) (Table 3). The most common dementia diagnosis was AD followed by VaD and DNOS (Table 1).

**Table 1:** Persons with dementia diagnosis, living at home or in a nursing home

Dementia diagnosis	Living at home n (%)^a^	Nursing home n (%)
Alzheimer’s disease	664 (55)	340 (43)
Alzheimer’s disease/Vascular dementia	72 (6)	45 (6)
Vascular dementia	200 (16)	164 (21)
Frontotemporal dementia	7 (1)	9 (1)
Lewy Body dementia	24 (2)	25 (3)
Dementia not otherwise specified (unknown)	193 (16)	170 (21)
Other dementia diagnosis	54 (4)	37 (5)

^a^Total of 1223 persons, missing = 9

**Table 2:** Persons with dementia not otherwise specified (DNOS) living at home

	Sweden *n* = 8	Finland *n* = 1	The NL *n* = 58	Germany *n* = 52	Estonia *n* = 42	France *n* = 4	Spain *n* = 16	The UK *n* = 12
Persons with DNOS, n (%) (total *n* = 193)	8 (4)	1 (1)	58 (30)	52 (26)	42 (21)	4 (2)	16 (8)	12 (6)
Age, years; mean (SD)	86 (6)	89 (0)	82 (6)	84 (6)	83 (9)	84 (4)	90 (7)	84 (6)
Gender, female (%)	6 (75)	0 (0)	32 (55)	37 (71)	31 (74)	3 (75)	13 (81)	6 (50)
Symptoms, years, mean (SD)	3.6 (3.7)	3 (3)	4.4 (2.7)	5.1 (3.6)	5.8 (5.0)	0.3 (0.5)	3.5 (2.1)	4.0 (3.6)
Comorbidity (CCI), *0*–37, mean, (SD)	2.1 (1.2)	2.0 (0)	2.0 (1.3)	2.8 (1.6)	2.6 (1.6)	2.3 (1.9)	2.6 (1.3)	1.9 (0.9)
Cognitive function (SMMSE), 0–*30*	20 (2)	23 (0)	14 (6)	13 (8)	12 (6)	14 (7)	14 (7)	17 (6)
Quality of life (QoL-AD), 13–*52*	36 (5)	27 (0)	35 (5)	35 (5)	28 (7)	–	34 (3)	35 (5)
Activities of daily living (Katz ADL), 0–*6*	4 (2)	4 (0)	3 (2)	2 (2)	3 (2)	6 (1)	2 (2)	5 (1)
Depression (CSDD), *0*–38, mean (SD)	8 (8)	13 (0)	7 (5)	11 (6)	11 (6)	3 (3)	10 (7)	8 (6)
Resource utilization (RUD) last 30 days (%)								
Hospital admission, geriatric ward	0	0	1 (2)	0	0	0	0	0
Visit general practitioner	3 (38)	0	29 (50)	42 (81)	9 (21)	4 (100)	7 (44)	1 (8)
Visit geriatrician	0	0	4 (7)	0	1 (2)	0	0	0
Visit neurologist	0	0	0	13 (25)	0	0	1 (6)	0
Visit psychiatrist	0	0	0	0	2 (5)	0	0	0
Neuropsychiatric symptoms (NPI-Q)								
Severity, *0*–36, mean, (SD)	11 (7)	12 (0)	10 (7)	11 (6)	12 (8)	5 (5)	8 (4)	8 (5)
Psychotropics, n (%)	4 (50)	1 (100)	1 (47)	33 (64)	24 (57)	2 (50)	14 (88)	7 (58)
Antipsychotics	0	0	13 (22)	16 (31)	18 (43)	0	7 (44)	2 (17)
Anxiolytic	0	0	7 (12)	3 (6)	2 (5)	2 (50)	5 (31)	0
Sedatives	1 (13)	1 (100)	1 (2)	4 (8)	4 (10)	1 (25)	2 (13)	0
Antidepressants	3 (38)	0	4 (7)	7 (14)	4 (10)	2 (50)	3 (19)	6 (50)
Anti-dementia medictation	2 (25)	1 (100)	10 (17)	21 (41)	2 (5)	0	5 (31)	2 (17)

Italicized values indicates a positive result, e.g., *0*–36

*p* ≤ 0.05 was regarded as significant; significant p-values are marked in bold

*AD* Alzheimer’s disease, *CCI* Charlson comorbidity index, *CSDD* Cornell scale for depression in dementia, *DNOS* Dementia not otherwise specified, *Katz ADL* Katz index of independence in activities of daily living, *n* Number of participants, *NL* The Netherlands, *NPI-Q* Neuropsychiatric inventory questionnaire, *QoL-AD* Quality of life in AD, *RUD* Recourse utilization in dementia questionnaire, *SMMSE* Standardized mini-mental state, *UK* United Kingdom, *VaD* Vascular dementia

**Table 3:** Persons with dementia not otherwise specified (DNOS) living in a nursing home

	Sweden *n* = 9	Finland *n* = 14	The NL *n* = 33	Germany *n* = 51	Estonia *n* = 22	France^a^ *n* = 0	Spain *n* = 28	The UK *n* = 13
Persons with DNOS, n (%), (total *n* = 170)	9 (5)	14 (8)	33 (19)	51 (30)	22 (13)	–	28 (17)	13 (8)
Age, years; mean (SD)	84 (3)	85 (7)	85 (6)	85 (7)	83 (7)	–	86 (8)	86 (7)
Gender, female (%)	6 (67)	8 (57)	28 (85)	34 (67)	18 (82)	–	23 (81)	10 (77)
Symptoms, years, mean (SD)	2.2 (1.5)	5.0 (2.4)	3.7 (2.6)	3.5 (3.1)	5.4 (6.8)	–	3.9 (4.6)	3.4 (2.9)
Comorbidity (CCI), *0*–37, mean, (SD)	1.9 (0.8)	3.3 (2.7)	2.2 (1.4)	2.8 (1.6)	2.7 (1.4)	–	2.0 (1.7)	2.1 (0.9)
Cognitive function (SMMSE), 0–*30*	13 (6)	16 (6)	10 (5)	14 (6)	10 (7)	–	12 (3)	13 (8)
Quality of life (QoL-AD), 13–*52*	37 (3)	29 (5)	35 (7)	34 (4)	27 (5)	–	30 (6)	34 (5)
Activities of daily living (Katz ADL), 0–*6*	3 (1)	2 (2)	2 (1)	2 (1)	2 (2)	–	1 (1)	3 (2)
Depression (CSDD), *0*–38, mean, (SD)	5 (4)	0 (0)	5 (5)	8 (5)	5 (4)	–	5 (4)	5 (3)
Resource utilisation (RUD) last 30 days, %						–		
Hospital admission, geriatric ward	0	0	0	0	0	–	0	0
Visit general practitioner	3 (34)	11 (79)	4 (12)	38 (75)	5 (23)	–	8 (29)	4 (31)
Visit geriatrician	1 (11)	1 (7)	1 (3)	0	0	–	14 (50)	0
Visit neurologist	0	0	0	26 (51)	0	–	4 (14)	0
Visit psychiatrist	0	0	0	0	5 (23)	–	2 (7)	1 (8)
Neuropsychiatric symptoms (NPI-Q)						–		
Severity, *0*–36	5 (7)	4 (4)	7 (6)	7 (6)	5 (5)	–	6 (6)	6 (4)
Psychotropics, use of yes/no, n (%)	7 (78)	9 (64)	24 (73)	35 (69)	13 (59)	–	24 (86)	10 (77)
Antipsychotics	1 (11)	2 (14)	16 (49)	19 (37)	10 (46)	–	16 (57)	4 (31)
Anxiolytic	2 (22)	3 (21)	5 (15)	5 (10)	1 (5)	–	9 (32)	0
Sedatives	2 (22)	5 (36)	6 (18)	3 (6)	1 (5)	–	7 (25)	0
Antidepressants	3 (33)	4 (29)	9 (27)	10 (20)	1 (5)	–	11 (39)	6 (46)
Antidementia	3 (33)	2 (14)	10 (30)	17 (33)	1 (5)	–	2 (7)	2 (15)

Italicized values indicates a positive result, e.g., *0*–36

*p* ≤ 0.05 was regarded as significant; significant p-values are marked in bold

*AD* Alzheimer’s disease, *CCI* Charlson comorbidity index, *CSDD* Cornell scale for depression in dementia, *DNOS* Dementia not otherwise specified, *Katz ADL* Katz index of independence in activities of daily living, *n* Number of participants, *NL* The Netherlands, *NPI-Q* Neuropsychiatric inventory questionnaire, *QoL-AD* Quality of life in AD, *RUD* Recourse utilization in dementia questionnaire, *SMMSE* Standardized mini-mental state, *UK* United Kingdom, *VaD* Vascular dementia

^a^values were missing in the French study

### Demographics of persons with DNOS living at home

The age of persons with DNOS living at home varied between 82 and 90 years and these persons were more often female (Table 2). Duration of symptoms in years and severity of neuropsychiatric symptoms was highest in Estonia and lowest in France. Comorbidity was highest in Germany and lowest in the United Kingdom. Cognitive function was highest in Finland and lowest in Estonia. Quality of life was highest in Sweden while the lowest QoL was found in Finland where the persons also had more depressive symptoms. Persons with DNOS in Germany and Spain were more dependent in ADLs while the highest proportion of independent persons were found in France and the United Kingdom. The use of anti-dementia medication was most frequent in Germany (Table 2). Regarding resource utilization during the last 30 days, persons with dementia living at home more commonly visited a GP (mean 40%) than a specialist (mean 19%).

### Demographics of persons with DNOS living in a nursing home

The persons with DNOS living in nursing homes were between 83 and 86 years of age and more often woman (Table 3). Duration of symptoms in years was highest in Estonia and lowest in Sweden. Comorbidity was highest in Finland and lowest in Sweden. Cognitive function was.

highest in Finland and lowest in Estonia and the Netherlands. Persons with DNOS in Sweden.

had the highest QoL score while the lowest QoL score was reported from Estonia. Persons with DNOS were less independent in Spain and more independent in Sweden and the United Kingdom. Depressive symptoms in this group was the lowest in Finland. Prevalence of neuropsychiatric symptoms was highest in the Netherlands and Germany and lowest in Finland. The use of anti-dementia medication was most frequent in Sweden and Germany and least frequent in Estonia (Table 3). Regarding resource utilisation during the last 30 days, persons with dementia living in a nursing home more often visited a GP (mean 49%) for medical advice than a specialist (mean 9%).

### Factors associated with DNOS

When comparing DNOS with other dementia diagnoses in persons living at home, bivariate regression analysis showed that factors associated with DNOS were higher age, more comorbid diseases, depressive symptoms and more severe neuropsychiatric symptoms (Table 4). Persons with DNOS living at home had more often visited a GP in outpatient care during the last 30 days, and had had fewer hospital admissions to geriatric wards and fewer visits to a geriatrician in outpatient care during the last 30 days. Lower use of psychotropics was associated with DNOS, specifically regarding treatment for anxiety, sleeping disorders, depression and dementia. In nursing homes, DNOS, compared with other dementia diagnoses, was associated with shorter duration of symptoms in years and with less severe neuropsychiatric symptoms. The DNOS group living in nursing homes also had lower use of psychotropic medication, especially regarding treatment for anxiety and dementia and more often visited a neurologist (Table 4).

**Table 4:** Bivariate regression analysis and associated factors of DNOS of persons with dementia

	Dementia diagnosis when living at home					Dementia diagnosis when living in a nursing home				
	DNOS *n* = 193	Other^a^ *n* = 1021	OR	CI 95%	*p* value	DNOS *n* = 170	Other^a^ *n* = 620	OR	CI 95%	*p* value
Person with dementia										
Age, years, median (Q1; Q3)	84 (80; 88)	82 (78; 86)	1.048	1.023–1.074	**< 0.001**	85 (80; 90)	84 (80; 88)	1.026	0.999–1.055	0.061
Gender, female %	66	63	1.163	0.840–1.608	0.363	75	74	1.053	0.714–1.555	0.793
Symptoms, years (range)	4 (2; 6)	4 (2; 6)	0.978	0.931–1.028	0.381	3 (1; 5)	4 (2; 7)	0.907	0.849–0.969	**0.004**
Comorbidity (CCI), median (Q1; Q3), *0*–37	2 (1; 3)	2 (1; 3)	–	–	**0.050**	2 (1; 3)	2 (1; 3)	–	–	0.994
Cognitive function (SMMSE), 0–*30*	15 (10; 19)	15 (10; 20)	0.987	0.963–1.011	0.284	13 (8; 17)	12 (7; 16)	1.023	0.992–1.055	0.154
Quality of life (QoL-AD), 13–*52*	34 (30; 38)	34 (30; 38)	0.996	0.965–1.027	0.789	33 (29; 36)	33 (28; 37)	0.996	0.962–1.030	0.802
Activities of daily living (Katz ADL), 0–*6*	3 (1; 5)	3 (2; 5)	–	–	0.098	2 (1; 3)	2 (1; 4)	–	–	0.397
Depression in dementia (CSDD), *0*–38	8 (5; 13)	7(3; 12)	1.032	1.006–1.059	**0.016**	5 (2; 9)	2 (2; 9)	0.991	0.956–1.027	0.611
Resource utilization (RUD) last 30 days, %										
Hospital admission, geriatric ward	1 (1)	42 (2)	0.121	0.017–0.887	**0.038**	0 (0)	1 (1)	0.000	0.000	1.000
Visit general practitioner	95 (48)	403 (40)	1.487	1.092–2.024	**0.012**	73 (43)	93 (47)	0.840	0.597–1.183	0.840
Visit geriatrician	5 (3)	75 (7)	0.335	0.134–0.841	**0.020**	17 (10)	55 (9)	1.139	0.643–2.020	0.655
Visit neurologist	14 (7)	44 (4)	1.737	0.932–3.235	0.082	30 (18)	43 (7)	2.875	1.741–4.748	**< 0.001**
Visit psychiatrist	2 (1)	2 (4)	0.435	0.102–1.856	0.261	8 (5)	27 (4)	1.083	0.483–2.429	0.847
Neuropsychiatric symptoms (NPI-Q)										
Severity, *0*–36	10 (6; 15)	8 (5; 14)	1.028	1.004–1.052	**0.021**	5 (2; 9)	6 (3; 11)	0.969	0.939–0.999	**0.043**
Psychotropics, n (%)	112 (58)	814 (80)	0.352	0.254–0.486	**< 0.001**	122 (72)	513 (83)	0.702	0.493–0.998	**0.049**
Antipsychotics	56 (29)	241 (24)	1.323	0.939–1.864	0.110	68 (40)	228 (37)	1.146	0.810–1.623	0.442
Anxiolytic	19 (10)	173 (17)	0.535	0.324–0.883	**0.014**	25 (15)	190 (31)	0.390	0.247–0.617	**< 0.001**
Sedatives	14 (7)	136 (13)	0.509	0.287–0.903	**0.021**	24 (14)	143 (23)	0.548	0.343–0.878	**0.012**
Antidepressants	29 (15)	330 (32)	0.370	0.244–0.561	**< 0.001**	44 (26)	175 (28)	0.888	0.604–1.305	0.546
Anti-dementia medication	43 (22)	585 (57)	0.214	0.149–0.307	**< 0.001**	37 (22)	271 (44)	0.358	0.241–0.533	**< 0.001**

Italicized values indicates a positive result, e.g., *0*–36

*p* ≤ 0.05 was regarded as significant; significant *p*-values are marked in bold

*AD* Alzheimer’s disease, *CCI* Charlson comorbidity index, *CI* Confidence interval, *CSDD* Cornell scale for depression in dementia, *FTD* Frontotemporal dementia, *Katz-ADL* Katz index of independence in activities of daily living, *LBD* Lewy body dementia, *n* Number of participants, *NL* The Netherlands, *NPI-Q* Neuropsychiatric inventory questionnaire, *OR* Odds ratio, *Q1* First quartile, *Q3* Third quartile, *QoL-AD* Quality of life in AD, *RUD* Resource utilization in dementia questionnaire, *SD* Standard deviation, *SMMSE* Standardized mini-mental state, *UK* United Kingdom, *VaD* Vascular dementia

^a^AD, AD/VaD/FTD/LBD/other

Associated factors in the multivariate regression analysis for DNOS compared with other dementia diagnoses in the eight European countries are summarized in Table 5. Factors associated with DNOS for persons living at home were younger age, more often visiting a GP during the last 30 days and lower use of medication for depression and dementia. In nursing homes, DNOS was associated with longer duration of symptoms in years, more often visiting a specialist physician (neurologist) during the last 30 days and lower use of anxiolytic and antidementia medication.

**Table 5:** Multivariate regression of associated factors for dementia not otherwise specified (DNOS)

Associated factors for DNOS	Adjusted R^2^	OR	95% CI	*P* value
Person with dementia				
Living at home (*n* = 193)	0.155			
Age		1.038	1.013–1.064	0.003
Resource utilization (RUD) last 30 days				
Visit general practitioner		1.672	1.207–2.316	0.002
Psychotropics				
Antidepressant		0.460	0.299–0.708	< 0.001
Anti-dementia medication		0.620	0.405–0.949	< 0.001
Living in a nursing home (*n* = 170)	0.160			
Symptoms, years		0.930	0.872–0.992	0.028
Resource utilisation (RUD) last 30 days				
Visit neurologist		4.151	2.055–8.383	< 0.001
Psychotropics				
Anxiolytic		0.448	0.258–0.780	0.005
Anti-dementia medication		0.307	0.185–0.509	< 0.001

*CI* Confidence interval, *OR* Odds ratio, *RUD* Resource utilization in dementia questionnaire

*p* ≤ 0.05 was regarded as significant

## Discussion

The prevalence of DNOS in the eight participating European countries was 16% in persons living at home and 21% in persons living in a nursing home. For persons living at home, DNOS compared with a specified dementia diagnosis, was associated with more severe neuropsychiatric symptoms and symptoms of depression with less anti-dementia or antidepressants medication. For persons living in a nursing home, DNOS was associated with more severe neuropsychiatric symptoms and less use of anti-dementia medication.

In neurocognitive disorders, a dementia diagnosis is essential for pharmacological and non-pharmacological treatment and for future care planning for people with dementia and their caregivers, as well as to increase the understanding and handling of problems like depression and BPSD for both the person and the formal and informal caregivers. In our study, independent of living condition, DNOS diagnoses were found in all the participating European countries with variation between 1 and 30% if living at home and between 5 and 30% if living in a nursing home. This variation may depend on differences in diagnostic procedures between European countries healthcare system and reimbursement structures as well as which professionals are involved. When analysing the healthcare and social service systems in the eight European countries participating in the RTPC project, “screening, diagnostic procedure, and treatment of dementia and complications” was however available for all/most people in six out of the eight countries with variation in professionals involved [34]. Another explanation for the variation in DNOS prevalence may be that countries have adapted differently to focus on a timely rather than early diagnosis as proposed by World Alzheimer International [2]. Another factor may be the accumulating scientific evidence for a high risk of adverse effects during treatment with acetylcholine esterase inhibitors such as nausea, vomiting, falls, bradycardia (donepezil) and with significant risk for deaths [5] that has led to some countries stopping reimbursement for these medications. Several countries have national guidelines or policies for dementia care and they also have standardized diagnostic and treatment procedures. Most people in European countries are referred to GPs to establish a dementia diagnosis, and some are referred to a specialist (neurologist or psychiatrist) [10]. In the present study, persons with DNOS living at home more often visited GPs (50%) than a specialists (namely a geriatrician in 3%, a neurologist in 7% and a psychiatrist in 1% of cases). These results are consistent with previous results [13] which also showed that people with a specific dementia diagnosis more often had visited a specialist (neurologist or psychiatrist). Hospital admission at a geriatric ward was significantly more common for persons with a specific dementia diagnosis compared to those with DNOS. This may indicate that with a known diagnosis, persons are more likely to be referred to professionals specializing in dementia. The result of this study showed that and anti-dementia medication was significantly lower for persons with DNOS, indicating that a specific diagnosis may be required for prescription of these medication. Other possible explanations are that the person was under investigation or had already tried anti-dementia medication and experienced side effects. Among those living in a nursing home, 43% of persons with DNOS visited a GP while a lower percentage visited a specialist (geriatrician 10%, neurologist 18% and psychiatrist 5%). Receiving a diagnosis of DNOS may depend on attitudes to dementia diagnosis and where the patient is diagnosed. For instance, outpatient care including primary care or specialised memory clinics differs between and within countries [13] and for this study we did not have any information about routines for cognitive testing in each facility but no significant differences in SMMSE suggesting the groups (DNOS and others) were comparable. Knowledge is sparse about the importance of a timely dementia diagnosis and what and when care interventions are needed in the course of the dementia disease and may affect the will or attitude to specify diagnosis. It seems important to systematically document when a dementia diagnosis is important for care and nursing and when the needs are more general.

In most European countries, pharmacological treatment with acetylcholinesterase inhibitors and/or memantine is available as treatment in AD. Anti-dementia medication in this study was used most frequently for treatment of people with DNOS in home care in Finland and Germany, and in nursing homes in Sweden and Germany. To our knowledge, studies that investigate DNOS differences and medical treatment between European countries are scarce. Inequalities may exist due to each country’s reimbursement policies that require decisions to be made by a specialist, e.g. differences have been found between France, the Netherlands, Spain and the United Kingdom [10]. Our findings also showed that AD was the most common dementia diagnosis and DNOS and VaD were the second most common diagnoses in the participating countries, which is consistent with previous findings [13]. As previously mentioned, a DNOS diagnosis was more common among people with dementia living in a nursing home than among people living in ordinary housing (mean 21 and 16%, respectively). This is similar to the results of previous studies (27 and 19%, respectively) [14, 15]. This may be due to professionals’ perception of the importance of a proper dementia diagnosis when a person has moved to a nursing home and also whether the physician responsible for medical treatment at the nursing home is a specialist or not. Similar to other studies [12] the prevalence of AD in this study was 55% in persons living at home and 43% in persons living in a nursing home and 22% were treated with acetylcholinesterase inhibitors and/or memantine. Timely diagnosis allows improved care planning and prognosis for the family [12] and should be prioritorized. Consequently, it should be possible to diagnose approximately 42–43% more people with AD, and enable them to get appropriate medical treatment if tolerated, care interventions and support when needed in the course of the dementia disease and in line with each country’s reimbursement structure.

When living at home, higher age seems to be associated with DNOS, as well as an increased risk of being depressed and having no adequate treatment. Our findings showed that age and depression were associated with DNOS. In a review by Lang et al. [16], undetected dementia was associated with age. There is uncertainty regarding the link between undetected dementia and comorbid depression but two studies found a possible link [16]. Previous studies have shown that late on-set depression is a risk factor for a dementia disease [35, 36], which makes it essential to diagnose the presence of and differentiate affective and cognitive symptoms and prescribe medical treatment as appropriate. Independent of dementia diagnosis, persons with a DNOS or other dementia diagnosis have similar QoL. Our study did not show any association between DNOS and comorbidity, nor cognitive function, QoL or ADL, when living in a nursing home even though lack of a specific dementia diagnosis has been suggested to reduce the persons QoL and independence [8]. A review by Cooper et al. [37] did not find any consistent evidence that pharmacological treatment improves QoL for people with dementia. People with dementia living at home has shown significantly higher QoL compared with people with dementia living in a nursing home [38]. This suggests that more studies are needed to investigate how QoL may be increased for persons with dementia living in a nursing homes.

One limitation of the study is that this is a secondary analysis of a more global study (RTPC) which was not aiming to study DNOS. The variable DNOS was determined when the type of dementia was “unknown”. Therefore, this category could include cases where, despite a thorough diagnostic procedure, the specialists could not define the type of dementia, as well as cases where no proper diagnosis procedure was applied. Therefore, the group of participants classified as “DNOS” may be heterogeneous. We investigated a large group of persons with dementia living at home (*n* = 1223) or in a nursing home (*n* = 790). In the bivariate regression the single factors (age, symptoms in years, resource utilization, psychotropic medication use and total scores) showed significance (*p* ≤ 0.05) with a small 95% confidence interval (CI) in most variables, suggesting that the sample was large enough. However, when investigating each country separately, some countries (Finland, France and Sweden), had very few participants with a DNOS and a large 95% CI which probably make the results less reliable in this respect. Therefore, only the results for the whole cohort are presented here.

All countries participating in the RTPC project used the same procedure and guidelines to establish internal validity. For external validity participating countries represented southern, northern, eastern, western and central Europe. The findings in this study should be interpreted with caution since one limitation is the cross sectional study design, and no causal relationship can be established. Moreover, the persons with dementia living at home were also a specific subgroup of persons with dementia, on the margin of care (e.g. when home care is not enough or when the caregiver is heavily burdened) and were judged to be at risk of institutional care within 6 months. This means that the participants may not be representative for persons with dementia in general.

## Conclusions

A diagnosis of DNOS is common but vary between countries. People with dementia who are older more often appear to have a DNOS diagnosis. They seem to be cared for more often by a GP, especially in home care, and receive fewer anxiolytics and antidepressant medications. Results of the bivariate analysis in the present study suggest that people with DNOS suffer from more severe neuropsychiatric symptoms, which would support the idea that a proper and specific diagnosis of dementia could help the management of their disease.

## Data Availability

The datasets used and/or analysed during the current study are available from the corresponding author on reasonable request.

## Abbreviations

AD: Alzheimer’s disease
ADLs: Activities of daily living
BPSD: Behavioural psychological symptoms in dementia
CCI: Charlson Comorbidity Index
CI: Confidence interval
CSDD: Cornell Scale for Depression in Dementia
DNOS: Dementia not otherwise specified
FTD: Frontotemporal dementia
Katz-ADL: Katz Index of Independence in Activities of Daily Living
LBD: Lewy body dementia
NL: The Netherlands
NPI-Q: Neuropsychiatric Inventory questionnaire
OR: Odds ratio
QoL: Quality of life
QoL-AD: Quality of Life in AD
RTPC: RightTimePlaceCare
RUD: Resource Utilization in Dementia
SD: Standard deviation
SMMSE: Standardized Mini-Mental State Examination
VaD: Vascular dementia
